# Trends and inequalities in oral rehydration therapy and continued feeding for children under five with diarrhoea in Sierra Leone

**DOI:** 10.1186/s41182-024-00633-0

**Published:** 2024-10-02

**Authors:** Augustus Osborne, Camilla Bangura

**Affiliations:** Department of Biological Sciences, School of Basic Sciences, PMB, Njala University, Freetown, Sierra Leone

**Keywords:** Children, Diarrhoea, Feeding, Public health, Sierra Leone

## Abstract

**Background:**

Sierra Leone has improved child health outcomes in recent decades. However, diarrhoeal diseases remain a public health concern, particularly among children under five. This study investigates the trends and inequalities in oral rehydration therapy and continued feeding for children under five with diarrhoea in Sierra Leone in 2008, 2013 and 2019.

**Methods:**

The analysis utilised data from the Sierra Leone Demographic Health Survey rounds conducted in 2008, 2013, and 2019. The software utilised for the calculation of various measures of inequality, including simple difference, ratio, population-attributable risk, and population-attributable fraction, was the World Health Organization Health Equity Assessment Toolkit. An inequality assessment was conducted for six stratifiers: maternal age, maternal economic status, maternal level of education, place of residence, sex of the child, and sub-national region.

**Results:**

Our findings reveal that children under five with diarrhoea receiving oral rehydration therapy and continued feeding increased from 56.5% in 2008 to 59.7% in 2019 in Sierra Leone. Children of mothers aged 20–49 had more coverage over time than those with mothers aged 15–19. Children of mothers who are wealthy, more educated, and living in urban areas show a decrease in coverage with time compared to the poor, the lowly educated, and those residing in rural areas. Male children had higher coverage than female children. Regional inequality decreased slightly from 21.5 percentage points in 2008 to 21.2 percentage points in 2019.

**Conclusion:**

The findings revealed a mixed picture of progress in oral rehydration therapy and continued feeding for children under five in Sierra Leone. While national coverage has increased, inequalities persist. Children of older mothers and those from disadvantaged backgrounds have experienced improvements, while children of younger, wealthier, and more educated mothers in urban areas have seen a decline in coverage. The gender and regional inequalities remain. Expanding community-based health programs, providing subsidised or free supplies, and strengthening health systems in underserved areas are key strategies to ensure equitable and effective healthcare for all children in Sierra Leone.

## Introduction

Diarrhoea is a common illness characterised by loose, watery stools occurring three or more times a day [1]. It is caused by various infectious agents, including viruses, bacteria, and parasites, often transmitted through contaminated food or water, poor sanitation, and inadequate hygiene practices [2]. In children under five, diarrhoea can lead to rapid dehydration, electrolyte imbalance, and malnutrition, all of which can be life-threatening [2, 3].

Diarrhoea remains a public health concern globally, particularly in low- and middle-income countries [4, 5]. According to the World Health Organization (WHO) and the United Nations Children's Fund (UNICEF), diarrhoeal diseases account for an estimated 525,000 deaths annually in children under five, with most of these deaths occurring in sub-Saharan Africa [6]. Sierra Leone is no exception with diarrhoea among the leading cause of childhood mortality in the country, with a substantial burden on the healthcare system [7]. According to the 2021 WHO statistics, diarrhoea accounts for 64.38 deaths per 100,000 under-fives in Sierra Leone [7]. This statistic highlights the critical public health issue of diarrhoeal diseases, often exacerbated by inadequate access to clean water, poor sanitation, and limited healthcare resources [4]. Addressing these underlying factors is essential to reducing the burden of diarrhoea and improving child health outcomes in Sierra Leone.

Interventions to combat childhood diarrhoea have been a global focus. The WHO and UNICEF recommend a multi-pronged approach, including access to safe water and sanitation, promotion of breastfeeding, and appropriate treatment with oral rehydration therapy (ORT) and continued feeding [8–10]. In collaboration with partner organisations, Sierra Leone's government has implemented various programmes to address diarrhoea by promoting the use of ORT to prevent dehydration in children. Vaccines, like the rotavirus vaccine, have reduced viral infections in infants. Access to clean water and sanitation facilities is also crucial. NGOs and government programmes are constructing latrines, promoting handwashing, and ensuring safe drinking water. Community-based health education programmes raise awareness about hygiene practices and ORT [11]. These campaigns have shown promising results, with coverage of ORT among children with diarrhoea increasing from 56.5% in 2008 to 59.7% in 2019 based on the results of our findings. However, challenges remain in ensuring consistent access to these interventions across the country, particularly in rural areas [11]. One major issue is the inadequate healthcare infrastructure, which often lacks essential facilities and trained personnel to provide timely and effective treatment [11]. Additionally, a shortage of clean water and sanitation facilities exacerbates the prevalence of waterborne diseases [12]. Cultural beliefs and practices can also hinder the acceptance of medical interventions, as some communities may rely on traditional remedies instead of seeking professional care [13]. Furthermore, logistical difficulties in delivering supplies, such as oral rehydration solutions and vaccines, to remote locations complicate efforts to implement consistent health programmes. These factors collectively impede the government's ability to ensure equitable access to necessary interventions, leaving vulnerable populations at greater risk.

Despite these efforts, a knowledge gap exists regarding the trends and inequalities in the utilisation of ORT and continued feeding practices among children under five with diarrhoea in Sierra Leone between 2008 and 2019, as previous studies have explored healthcare seeking in children and the prevalence and risk factors of diarrhoel diseases in adults’ population [12, 13]. This study aims to address this gap by analysing national data to identify trends in treatment practices over time and potential disparities based on geographic region and socioeconomic status. Understanding these trends and inequalities is crucial for informing targeted interventions and improving child health outcomes in Sierra Leone.

## Methods

### Study setting and data source

Data were obtained from the 2008, 2013, and 2019 Sierra Leone Demographic Health Survey (SLDHS). The SLDHS is a nationwide survey that aims to identify consistent patterns and changes in demographic indicators, health indicators, and social issues among individuals of all genders and age groups. The SLDHS had a cross-sectional design in which participants were chosen using a stratified multi-stage cluster sampling method. The SLDHS report provides detailed information on the sampling methodology [14]. This study involved children under five with a history of use of ORT with continued feeding for treating childhood diarrhoea. The 2008, 2013, and 2019 SLDHS data were available through the WHO HEAT online platform [15]. This study considered the parameters specified in the Guidelines for Strengthening the Reporting of Observational Studies in Epidemiology (STROBE) [16].

### Variables and measures

The outcome variable was ORT and continued feeding to treat childhood diarrhoea. The calculation involved determining the proportion of children under five who used ORT with continued feeding for treating childhood diarrhoea. The six available variables in the WHO HEAT online software disaggregation for children aged < 5 years with diarrhoea receiving oral rehydration therapy and continued feeding in Sierra Leone were utilised for the data analysis: mother’s age, maternal education, place of residence, economic status, sex of the child, and sub-national regions. The age groups were classified as 15–19 and 20–49. The maternal educational status was categorised as no education, primary, secondary or higher education. This study classified economic status into five categories: poorest, poor, middle, rich, and richest. Residential areas were classified as either urban or rural. The sub-national regions encompassed the geographical areas of East, North, Northwestern, South, and West.

### Statistical analyses

The analyses were performed using the online HEAT software developed by the World Health Organization. We conducted data analysis using four summary metrics of health inequity: Difference (D), Ratio (R), Population Attributable Risk (PAR), and Population Attributable Fraction (PAF). The four specified metrics are classified as simple (D, R), complex (PAR, PAF), relative (R, PAF), and absolute (D, PAR). Following the World Health Organization’s suggestion that policy-relevant findings require a range of summary indicators, we employed these multiple metrics. Refer to other references for more comprehensive guidance on calculating these summary metrics [17, 18]. In our current study, we determined D by subtracting the percentage of use of ORT and continued feeding males from females, the percentage of the poorest group from the richest group, the percentage of individuals with no education from those with secondary/higher education, and the percentage of individuals in rural areas from those in urban areas. These calculations were performed for the variables of sex of the child, wealth quintile, education, and residence, respectively. About the sub-national region, we computed D as the difference between the region with the greatest estimate for each survey round and the regions with the lowest estimate for each survey. Regarding the place of residency, the value of R was determined by dividing the size of the higher subpopulation (Yhigh) by the size of the lower subpopulation (Ylow), expressed as R = Yhigh/Ylow. Yhigh refers to those residing in urban areas, whilst Ylow refers to those in rural areas. Regarding education, Yhigh and Ylow represented the most privileged group (i.e. women with secondary or higher education) and the most disadvantaged group (those without any education), respectively. Similarly, Yhigh corresponded to the highest wealth quintile, whereas Ylow corresponded to the lowest wealth quintile. Yhigh denotes the ratio of male children who have had complete immunisation, while Ylow denotes the ratio of female children who have received complete immunisation. The PAR values were calculated by subtracting the estimated values for the reference categories (i.e. yref) from the national average of the use of ORT with continued feeding among under-fives. Hence, equation (l): PAR = yref-l represents the correlation between the performance indicator PAR and the national average of full vaccination coverage. The PAF is a metric that quantifies the level of inequality in the PAR. It is calculated using the formula PAF = (PAR/ l)*100. In addition, the 95% confidence intervals (CIs) were calculated for the point estimations. A significant disparity arises when D and PAR's lower and upper bounds do not include zero.

## Results

**Fig. 1:**
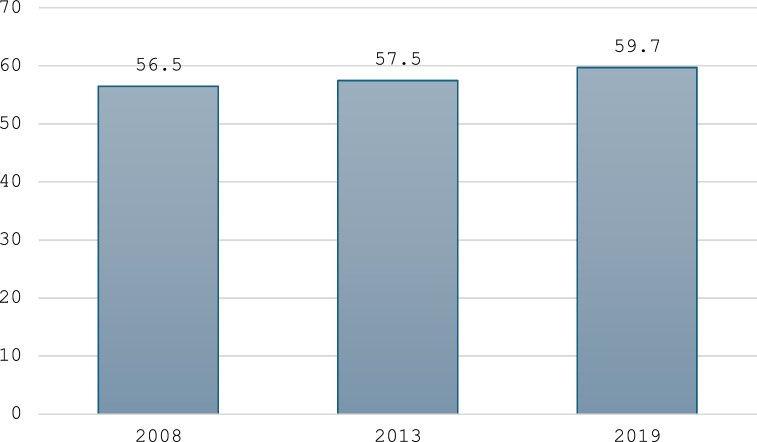
Prevalence of children aged < 5 years with diarrhoea receiving oral rehydration therapy and continued feeding in Sierra Leone in 2008, 2013 and 2019

Figure 1 shows the prevalence of children aged < 5 years with diarrhoea receiving oral rehydration therapy and continued feeding in Sierra Leone in 2008, 2013 and 2019. Nationally, ORT coverage increased from 56.5% in 2008 to 59.7% in 2019.

### Trends in the prevalence of children aged < 5 years with diarrhoea receiving oral rehydration therapy and continued feeding by different inequality dimensions in Sierra Leone, 2008, 2013 and 2019

Table 1 shows the trends in children aged < 5 years with diarrhoea receiving oral rehydration therapy and continued feeding in Sierra Leone in 2008, 2013 and 2019. For children of mothers aged 20–49 years, ORT coverage rose from 56.1% in 2008 to 60.8% in 2019. Children of mothers from the poorest quintile (Quintile 1) saw an increase in ORT coverage from 46.6% in 2008 to 57.7% in 2019. Children with mothers without education experienced an increase in ORT coverage from 55.4% in 2008 to 63.1% in 2019. Children with mothers from rural areas showed an increase in ORT coverage from 55.6% in 2008 to 60.3% in 2019. Children with mothers from the North region had the highest coverage, from 63.9% in 2008 to 70.5% in 2019. For children of mothers aged 15–19, ORT coverage decreased from 61.7% in 2008 to 42.9% in 2019. Children with mothers of the richest quintile (Quintile 5) showed a slight decrease in ORT coverage from 60.2% in 2008 to 56.4% in 2019. Children of mothers with primary education experienced a decrease in ORT coverage from 67.2% in 2008 to 59.6% in 2019. Children of mothers with secondary or higher education groups decreased from 49.4% in 2008 to 52.9% in 2019. Children with mothers living in urban areas had a decline in ORT coverage from 59.4% in 2008 to 58.5% in 2019. Females slightly decreased ORT coverage from 57.9% in 2008 to 57.5% in 2019. Children with mothers living in the East region had the lowest coverage, which increased from 42.3% in 2008 but remained relatively low at 56.4% in 2019.

**Table 1:** Trends in the prevalence of children aged < 5 years with diarrhoea receiving oral rehydration therapy and continued feeding by different inequality dimensions in Sierra Leone, 2008, 2013, and 2019

	2008				2013				2019			
Dimension	N	%	LB	UB	N	%	LB	UB	N	%	LB	UB
Age												
15–19 years	46	61.7	45.4	75.7	105	51	39	63	39	42.9	26.4	61.2
20–49 years	629	56.1	50.2	61.7	1095	58.1	54.2	61.9	590	60.8	55.6	65.7
Economic status												
Quintile 1 (poorest)	148	46.6	35.8	57.7	266	57.6	50.3	64.6	136	57.7	49.2	65.8
Quintile 2	162	57.6	46.8	67.7	267	60.0	53.5	66.3	160	65.3	56.5	73.1
Quintile 3	161	57.9	47.6	67.5	250	54.1	46.0	62.0	127	55.5	44.9	65.7
Quintile 4	130	62.4	49.5	73.8	234	56.0	48.3	63.4	117	61.2	49.6	71.7
Quintile 5 (richest)	72	60.2	49.2	70.2	180	60.5	50.9	69.3	87	56.4	43.5	68.4
Education												
No education	513	55.4	48.6	62.0	833	58.2	53.7	62.5	339	63.1	56.5	69.3
Primary education	94	67.2	55.5	77.1	168	54.1	44.8	63.2	121	59.6	49.3	69.0
Secondary or higher education	67	49.4	35.9	63.0	199	57.6	49.4	65.4	169	52.9	44.5	61.2
Residence												
Rural	523	55.6	48.6	62.4	879	56.9	52.6	61.2	412	60.3	54.5	65.8
Urban	152	59.4	49.6	68.5	321	59.1	53.2	64.8	217	58.5	49.4	67.0
Sex of the child												
Female	326	57.9	49.6	65.8	610	55.8	51.3	60.2	288	57.5	50.9	63.9
Male	349	55.1	48.5	61.5	590	59.3	54.2	64.3	341	61.5	54.6	68.0
Region												
East	121	42.3	31.6	53.8	239	55.7	47.7	63.4	163	56.4	47.5	65.0
North	371	63.9	55.5	71.5	576	56.8	51.8	61.6	70	70.5	58.3	80.3
Northwestern	NA	NA	NA	NA	NA	NA	NA	NA	154	66.6	55.7	76.0
South	104	44.6	31.9	58.2	211	58.6	49.0	67.6	113	49.3	39.1	59.4
West	78	58.9	48.5	68.6	173	61.3	52.5	69.4	127	58.7	46.0	70.3

*N* sample size, *%* percentage, *LB* lower bound, *UB* upper bound, *NA* not available as between 2008 and 2013, Sierra Leone had four regions

### Regional prevalence of children aged < 5 years with diarrhoea receiving oral rehydration therapy and continued feeding in Sierra Leone in 2019

**Fig. 2:**
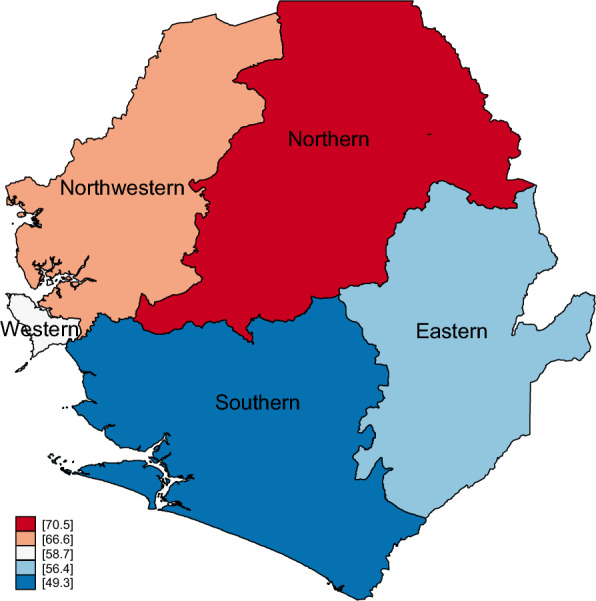
Regional prevalence of children aged < 5 years with diarrhoea receiving oral rehydration therapy and continued feeding in Sierra Leone in 2019

Figure 2 shows the regional prevalence of children aged < 5 years with diarrhoea receiving oral rehydration therapy and continued feeding in Sierra Leone in 2019. The northern region had the highest prevalence of 70.5% of children aged < 5 years with diarrhoea receiving oral rehydration therapy and continued feeding in Sierra Leone, whilst the southern region had the lowest prevalence of 49.3% of children aged < 5 years with diarrhoea receiving oral rehydration therapy and continued feeding in Sierra Leone.

### Inequality measures of estimates of factors associated with children aged < 5 years with diarrhoea receiving oral rehydration therapy and continued feeding in Sierra Leone, 2008, 2013 and 2019

Table 2 presents the inequality measures for children aged < 5 years with diarrhoea receiving oral rehydration therapy and continued feeding in Sierra Leone from 2008, 2013 and 2019. Age inequality increased from -5.6 percentage points in 2008 to 17.8 percentage points in 2019. The PAR indicates that the setting average could have been 0 percentage points higher in 2008, 0.5 percentage points higher in 2013, and 1.1 percentage points higher in 2019 without age inequality. Economic inequality increased from 13.5 percentage points in 2008 to 14.6 percentage points in 2019. The PAR suggests that the setting average could have been 3.7 percentage points higher in 2008 and 2.9 percentage points higher in 2013; however, it was zero in 2019, indicating no further improvement could be achieved. Child's sex inequality increased from -2.8 percentage points in 2008 to 4.0 percentage points in 2019. The PAR reveals that the setting average was 0 in 2008, 1.8 percentage points higher in 2013 and 2019 without considering the child's sex. Educational inequality decreased from -6.0 percentage points in 2008 to -10.1 percentage points in 2019. The PAF and PAR were zero in 2008, 2013, and 2019, indicating that no further improvement could be achieved. Place of residence inequality decreased from 3.7 percentage points in 2008 to -1.7 percentage points in 2019. The PAR indicates that the setting average could have been 2.9 percentage points higher in 2008 and 1.6 percentage points higher in 2013; however, it was zero in 2019, indicating no further improvement could be achieved. Regional inequality decreased slightly from 21.5 percentage points in 2008 to 21.2 percentage points in 2019. The PAR reveals that the setting average could have been 7.4 percentage points higher in 2008, 3.7 percentage points higher in 2013, and 10.8 percentage points higher in 2019 without regional inequality.

**Table 2:** Inequality measures of estimates of factors associated with children aged < 5 years with diarrhoea receiving oral rehydration therapy and continued feeding in Sierra Leone, 2008, 2013, and 2019

	2008			2013			2019		
Dimension	Est	LB	UB	Est	LB	UB	Est	LB	UB
Age									
D	−5.6	−22.1	10.9	6.5	−6.3	19.3	17.8	−0.8	36.6
PAF	0	−0.0	0.0	0.9	0.9	1.0	1.8	1.8	1.8
PAR	0	−0.9	0.9	0.5	−0.3	1.4	1.1	0.0	2.1
R	0.9	0.6	1.1	1.1	0.8	1.4	1.4	0.9	2.1
Economic status									
D	13.5	−1.7	28.8	2.8	−8.8	14.6	−1.3	−16.5	13.8
PAF	6.5	6.4	6.7	5.0	4.9	5.2	0	−0.1	0.1
PAR	3.7	−6.8	14.3	2.9	−3.6	9.5	0	−9.6	9.6
R	1.2	0.9	1.7	1.0	0.8	1.2	0.9	0.7	1.2
Education									
D	−6.0	−21.3	9.3	−0.5	−9.7	8.5	−10.1	−20.7	0.3
PAF	0	−0.2	0.2	0.1	0.0	0.2	0	−0.1	0.1
PAR	0	−11.3	11.3	0.0	−6.1	6.3	0	−6.4	6.4
R	0.8	0.6	1.2	0.9	0.8	1.1	0.8	0.6	1.0
Residence									
D	3.7	−7.9	15.5	2.2	−4.9	9.4	−1.7	−12.3	8.7
PAF	5.1	5.0	5.3	2.8	2.7	2.8	0	−0.0	0.0
PAR	2.9	−3.9	9.8	1.6	−2.9	6.2	0	−5.2	5.2
R	1.0	0.8	1.3	1.0	0.91	1.1	0.9	0.8	1.1
Sex of the child									
D	−2.8	−13.2	7.5	3.5	−3.1	10.2	4.0	−5.3	13.3
PAF	0	−0.0	0.0	3.1	3.0	3.1	3.0	3.0	3.1
PAR	0	−3.6	3.6	1.8	−1.0	4.6	1.8	−1.6	5.3
R	0.9	0.7	1.1	1.0	0.9	1.1	1.0	0.9	1.2
Region									
D	21.5	7.7	35.3	5.5	−6.0	17.2	21.2	6.1	36.2
PAF	13.1	13.1	13.2	6.5	6.4	6.6	18.1	17.9	18.2
PAR	7.4	3.9	10.9	3.7	−2.9	10.5	10.8	0.6	20.9
R	1.5	1.1	2.0	1.1	0.9	1.3	1.4	1.1	1.8

*Est* estimates, *LB* lower bound, *UB* upper bound

## Discussion

This study investigates trends and inequalities in using ORT with continued feeding for treating childhood diarrhoea in Sierra Leone between 2008, 2013 and 2019. While national coverage for ORT with continued feeding remained relatively stagnant, there was a concerning widening of inequalities in access across different groups.

The study reveals a modest increase in the percentage of children under five years old with diarrhoea receiving oral rehydration therapy and continued feeding, rising from 56.5% in 2008 to 59.7% in 2019. This increase is a positive indicator of progress in addressing childhood diarrhoea, a leading cause of morbidity and mortality in young children worldwide. Several factors may have contributed to this increase in ORT coverage. Enhanced public health campaigns, community health education, and increased availability of ORT solutions at health facilities could play pivotal roles [19]. For instance, initiatives to educate mothers about the importance of ORT and continued feeding during diarrhoea episodes can lead to improved health-seeking behaviours [7, 20]. Additionally, the increase aligns with broader efforts to strengthen health systems in Sierra Leone, particularly following the Ebola outbreak, which emphasised the need for robust healthcare infrastructure [11]. Investments in maternal and child health programmes may also have contributed to this positive trend [11]. Our finding is closely linked to Sustainable Development Goals (SDGs) 3 target 3.2, which aims to end preventable deaths of newborns and children under five years of age, which is directly supported by effective treatment of diarrhoea through ORT. As Sierra Leone continues to work towards achieving the SDGs, particularly those related to health, this finding highlights both progress made and the challenges in improving child health outcomes. Mothers aged 20–49 had higher rates of ORT with continued feeding for their children with diarrhoea in Sierra Leone. This finding is consistent with the previous studies [4, 21]. Mothers aged 20–49 might have greater awareness about ORT and continued feeding due to living longer and potentially having more experience caring for children [22]. Mothers in the 20–49 age range might be more likely to have had access to healthcare information or attended prenatal care visits where they could have learned about ORT and continued feeding compared to younger mothers [23]. Younger mothers (15–19) might face more social or economic challenges, making it harder to access healthcare facilities or follow treatment recommendations. This could be due to a lack of transportation, childcare for other children, or feeling less comfortable navigating the healthcare system [24].

Our study revealed that children of wealthy and more educated mothers show a decrease in coverage of ORT with continued feeding with time compared to the poor and the lowly educated in Sierra Leone. This finding suggests that while overall access to interventions may improve, the benefits are not equally distributed [25]. Wealthier families, who might have greater access to information and resources, may be less reliant on public health initiatives or may prioritising different health practices, potentially leading to a decline in the uptake of essential interventions like ORT. In contrast, poorer and less educated families, who often face more significant barriers to healthcare access, may increasingly rely on these critical interventions as they become more aware of their importance in managing diarrhoea [26]. This disparity raises essential questions about the effectiveness of health education and outreach programmes in reaching all population segments. It underscores the need for targeted strategies that specifically address the needs of wealthier and more educated families, ensuring they remain engaged with public health initiatives [27]. Additionally, it highlights the necessity of fostering community-level support and education to reinforce the importance of ORT and continued feeding, particularly in urban areas where the decline is most pronounced [28–30]. These findings are closely linked to SDG 3 (Good Health and Well-Being) and SDG 10 (Reduced Inequality). The observed decrease in coverage among wealthier populations indicates a potential setback in achieving SDG 3, as equitable access to health interventions is crucial for reducing child mortality rates. Moreover, the disparities highlighted in our study resonate with SDG 10, which seeks to reduce inequality within and among countries. The findings suggest that without targeted interventions, health inequities may persist or even widen, particularly between different socioeconomic groups. Addressing these disparities is essential for improving child health outcomes, fostering social equity and ensuring that all children, regardless of socioeconomic status, have access to life-saving interventions.

Our study revealed that male children gained more access to ORT and continued feeding treatment than female children in Sierra Leone. This finding aligns with the previous study [4]. In some cultures, with a son preference, boys might receive more attention and resources for healthcare compared to girls. This could make girls with diarrhoea less likely to receive ORT and continued feeding [31]. Traditionally, healthcare decisions for children might lie with fathers or male heads of households. These individuals might hold different beliefs about treatment priorities for sons versus daughters, impacting access to ORT and continued feeding [32]. Further investigation is needed to understand the specific reasons behind the widening gender gap in ORT and continued feeding access.

Our study reveals a troubling trend as children of mothers in urban areas of Sierra Leone exhibit a decrease in coverage of oral rehydration therapy with continued feeding over time, in contrast to their rural counterparts. This finding suggests that despite the perception that urban areas may have better access to healthcare resources, this is not translating into improved health outcomes for children suffering from diarrhoea. Several factors may contribute to this decline. Urban families might experience a shift in health-seeking behaviour, potentially relying more on private healthcare options or alternative remedies rather than public health interventions [33]. Additionally, rapid urbanisation and associated lifestyle changes may lead to increased stress and less emphasis on traditional health practices, impacting the uptake of essential interventions like ORT [34]. The decrease in ORT coverage among urban populations also raises concerns about the effectiveness of health education and outreach programmes in these areas. It highlights the necessity for tailored interventions that address the unique challenges faced by urban families, such as misinformation about health practices or a lack of engagement with public health initiatives. Moreover, as urban settings often experience higher rates of migration and diversity, targeted community-based programmes that emphasise the importance of ORT and continued feeding are essential to ensure that all families, regardless of their background, understand and utilise these critical health interventions. These findings are closely aligned with SDG 3 and SDG 11. The observed decline in ORT coverage among urban populations indicates a potential barrier to achieving SDG 3, emphasising the need for equitable access to health interventions across different living environments. Furthermore, our findings relate to SDG 11, which focuses on making cities and human settlements inclusive, safe, resilient, and sustainable. As urban areas grow, ensuring that health services are accessible and effective becomes increasingly critical. Addressing the decline in ORT coverage in urban settings is essential for fostering healthier communities and reducing health disparities.

The regional disparities in ORT and continued feeding for childhood diarrhoea remained high in Sierra Leone despite a slight downward trend. Although the overall picture might be improving, there could be a persistent gap in the distribution of healthcare facilities, trained personnel, and ORT supplies across different regions [35]. Remote or underserved areas might still have limited access to these resources, hindering proper treatment for diarrhoea. Poor infrastructure in certain regions, like limited transportation networks, could make it difficult for families to reach healthcare facilities, especially during emergencies or for follow-up care, impacting their ability to consistently adhere to ORT and continued feeding practices [36]. Addressing these potential reasons requires a multi-pronged approach. Investing in infrastructure development, ensuring equitable distribution of healthcare resources, and tailoring public health interventions to address regional needs are crucial steps. Engaging with communities and promoting health literacy can further bridge the gap and ensure all children in Sierra Leone have access to effective treatment for diarrhoea.

### Policy and practice implications

Our study on children under five with diarrhoea receiving oral rehydration therapy and continued feeding in Sierra Leone in 2008, 2013, and 2019 reveals the need for policy and healthcare practices adjustments. Policies promoting ORT and continued feeding should prioritise addressing identified inequalities. This could involve targeted campaigns, resource allocation based on need, and collaboration with local community leaders. Develop age-appropriate education materials and outreach programmes to ensure caregivers understand the specific needs of different age groups during diarrhoea episodes. Explore policy solutions like subsidies for ORT or financial assistance programmes to improve access for underprivileged communities. Integrate diarrhoea treatment best practices into existing educational programmes for mothers and caregivers. This could involve collaborating with the Ministry of Education or incorporating these topics into antenatal care services. Further research is needed to understand the reasons behind the widening sex disparity. Policy interventions could then address these specific cultural or social barriers. Allocate resources and healthcare worker training based on identified regional inequality. Consider implementing pilot programmes in high-burden regions to test and refine targeted interventions. Regularly collect treatment coverage and inequalities data to track progress and identify areas where policies and practices need further refinement. By implementing these policy and practice changes, Sierra Leone can work towards achieving equitable access to effective diarrhoea treatment for all children under 5, regardless of age, economic background, education level, residence, sex, or region.

### Strengths and limitations

The SLDHS provide nationally representative data, allowing for generalisable conclusions about trends and inequalities across the country. SLDHS likely include variables on childhood diarrhoea, ORT use, continued feeding practices, child age, economic status, maternal education, place of residence, child’s sex, and region. These are all directly relevant to the research question. WHO Heat is specifically designed to analyse and visualise health survey data. This can be a strength as it simplifies data analysis, creates publication-quality visualisations, and facilitates the calculation of concentration indices and population-attributable fractions used to assess inequality. While SLDHS follow standardised protocols, recall or social desirability bias in survey responses can still affect data quality. SLDHS data may not capture all the nuances of care practices. For instance, they may not differentiate between types of ORT used or provide details on how continued feeding was practised. Ecological fallacy is a potential concern since the data are collected at the household level. This means conclusions drawn about individuals may not be accurate.

## Conclusion

Despite a slight increase in national coverage of oral rehydration therapy and continued feeding for children under five in Sierra Leone, significant disparities persist. While children of older mothers and those from disadvantaged backgrounds have shown improvements, the coverage for children of younger, wealthier, and more educated mothers in urban areas has declined. The gender and regional inequalities remain pronounced. The government should develop age-appropriate education campaigns and outreach programmes to ensure proper diarrhoea treatment for all age groups of mothers of children under. Address the economic barriers to accessing adequate treatment by implementing financial assistance programmes or subsidised ORT solutions. Utilise existing educational structures to promote best practices for diarrhoea treatment in children. Investigate and address the underlying reasons behind the widening sex disparity in treatment access. Implement targeted interventions in regions with persistently high disparity in treatment access. Regularly monitor trends in coverage and inequalities to track progress and identify areas for further improvement. By implementing these recommendations, Sierra Leone can strive towards achieving equitable and optimal treatment for all children suffering from diarrhoea.

## Data Availability

The dataset used can be accessed at https://whoequity.shinyapps.io/heat/

## Abbreviations

D: Difference
HEAT: Health equity assessment toolkit
DHS: Demographic health survey
ORT: Oral rehydration therapy
PAF: Population attributable fraction
PAR: Population attributable risk
R: Ratio
SDG: Sustainable development goal
WHO: World Health Organization

## References

[1] Olopha OO, Egbewale B. Awareness and knowledge of diarrhoeal home management among mothers of under-five in Ibadan Nigeria. Univ J Public Health. 2017;5(1):40–5.

[2] Moore SR, Lima NL, Soares AM, Oriá RB, Pinkerton RC, Barrett LJ, Guerrant RL, Lima AA. Prolonged episodes of acute diarrhoea reduce growth and increase the risk of persistent diarrhoea in children. Gastroenterology. 2010;139(4):1156–64. 20638937 10.1053/j.gastro.2010.05.076 PMC2949449

[3] Troeger C, Colombara DV, Rao PC, Khalil IA, Brown A, Brewer TG, Guerrant RL, Houpt ER, Kotloff KL, Misra K, Petri WA. Global disability-adjusted life-year estimates of long-term health burden and undernutrition attributable to diarrhoeal diseases in children younger than five years. Lancet Glob Health. 2018;6(3):e255–69. 29433665 10.1016/S2214-109X(18)30045-7 PMC5861379

[4] Seifu BL, Legesse BT, Yehuala TZ, Kase BF, Asmare ZA, Mulaw GF, Tebeje TM, Mare KU. Factors associated with the co-utilisation of oral rehydration solution and zinc for treating diarrhoea among under-five children in 35 sub-Saharan Africa countries: a generalised linear mixed effect modelling with robust error variance. BMC Public Health. 2024;24(1):1329. 38755544 10.1186/s12889-024-18827-w PMC11100298

[5] UNICEF. Oral Rehydration Salts and Zinc - Market and Supply Update September. 2022. https://www.unicef.org/supply/media/13851/file/ORS-and-Zinc-Market-Supply-Update-September-2022.pdf Accessed 29 June 2024.

[6] WHO. Diarrhoeal disease. 2017. https://www.who.int/en/news-room/fact-sheets/detail/diarrhoeal-disease. Accessed 29 June 2024.

[7] World Health Organisation. Global health estimates: Leading causes of death. Who.int. 2021.https://www.who.int/data/gho/data/themes/mortality-and-global-health-estimates/ghe-leading-causes-of-death. Accessed 29 June 2024.

[8] Boschi-Pinto C, Labadie G, Dilip TR, Oliphant N, Dalglish SL, Aboubaker S, Agbodjan-Prince OA, Desta T, Habimana P, Butron-Riveros B, Al-Raiby J. Global implementation survey of Integrated Management of Childhood Illness (IMCI): 20 years on. BMJ open. 2018;8(7):e019079. 30061428 10.1136/bmjopen-2017-019079 PMC6067364

[9] Plan GA. Ending Preventable Child Deaths from Pneumonia and Diarrhoea by 2025 The Integrated Global Action Plan for Pneumonia and Diarrhoea (GAPPD). 2012. https://www.who.int/publications/i/item/9789241505239. Accessed 29 June 2024. 10.1136/archdischild-2013-305429 25613963

[10] Black R, Fontaine O, Bhan M, Huicho L, Arifeen E, Masanja H et al. Drivers of the reduction in childhood diarrhoea mortality 1980–2015 and interventions to eliminate preventable diarrhoea deaths by 2030. 2019;9(2):1–9. 10.7189/jogh.09.020801 PMC6815873 31673345

[11] Ministry of Health and Sanitation – (MoHS) Sierra Leone .2019.https://mohs.gov.sl/. Accessed 29 June 2024.

[12] Bah D, Gebru G, Hakizimana JL, Ogbonna U, Sesay B, Bah B, Mansaray P, Charles J, Jimmy A, Leno A, Jalloh F. Prevalence and risk factors of diarrheal diseases in Sierra Leone, 2019: a cross-sectional study. Pan Afr Med J. 2022;41:1. 10.11604/pamj.2022.41.3.32403 PMC8797046 35145595

[13] Diaz T, George AS, Rao SR, Bangura PS, Baimba JB, McMahon SA, Kabano A. Healthcare seeking for diarrhoea, malaria and pneumonia among children in four poor rural districts in Sierra Leone in the context of free health care: results of a cross-sectional survey. BMC Public Health. 2013;13:1–2. 23425576 10.1186/1471-2458-13-157 PMC3598532

[14] Statistics Sierra Leone (Stats SL) and ICF. Sierra leone demographic and health survey 2019. Freetown: Sierra Leone; 2020.

[15] World Health Organization. Health equity assessment toolkit plus (heat plus): software for exploring and comparing health inequalities in countries. Upload database edition. Geneva: World Health Organization; 2024.

[16] Von Elm E, Altman DG, Egger M, Pocock SJ, Gøtzsche PC, Vandenbroucke JP. The strengthening the reporting of observational studies in epidemiology (STROBE) statement: guidelines for reporting observational studies. Int J Surg. 2014;12(12):1495–9. 25046131 10.1016/j.ijsu.2014.07.013

[17] World Health Organization. Handbook on health inequality monitoring: with a special focus on low-and-middle-income countries. Geneva: World Health Organization; 2013.

[18] Hosseinpoor AR, Nambiar D, Schlotheuber A, Reidpath D, Ross Z. Health equity assessment toolkit (heat): software for exploring and comparing health inequalities in countries. BMC Med Res Methodol. 2016;16(1):1–10. 27760520 10.1186/s12874-016-0229-9 PMC5069829

[19] Olson CK, Blum LS, Patel KN, Oria PA, Feikin DR, Laserson KF, Wamae AW, Bartlett AV, Breiman RF, Ram PK. Community case management of childhood diarrhoea in a setting with declining use of oral rehydration therapy: findings from cross-sectional studies among primary household caregivers, Kenya, 2007. Am J Trop Med Hyg. 2011;85(6):1134. 22144458 10.4269/ajtmh.2011.11-0178 PMC3225166

[20] Lokossou YU, Tambe AB, Azandjèmè C, Mbhenyane X. Socio-cultural beliefs influence feeding practices of mothers and their children in Grand Popo, Benin. J Health Popul Nutr. 2021;40:1–2. 34301341 10.1186/s41043-021-00258-7 PMC8299590

[21] Fagbamigbe AF, Joseph J, Odongo AO, Mogere D, Kariuki J. Evaluation of the awareness and utilisation of oral rehydration salt and zinc in managing diarrhoea among under-five children in Oyo State, Nigeria. Int J Commun Med Pub Health. 2022;9(7):2785.

[22] Setorglo J, Klevor MK, Gorleku PN, Asomboy M, Kwadwo K, Pereko A, Adobasom-Anan AG, Steiner-Asiedu M. Mothers/Caregivers Age and Family Structure Predicted Knowledge on Recommended Nutrition Practices for Children under 5 Years.

[23] Bello CB, Esan DT, Akerele SA, Fadare RI. Maternal health literacy, utilisation of maternal healthcare services and pregnancy outcomes among newly delivered mothers: A cross-sectional study in Nigeria. Public Health in Practice. 2022;1(3): 100266. 10.1016/j.puhip.2022.100266 PMC9461586 36101756

[24] Grand-Guillaume-Perrenoud JA, Origlia P, Cignacco E. Barriers and facilitators of maternal healthcare utilisation in the perinatal period among women with social disadvantage: a theory-guided systematic review. Midwifery. 2022;1(105): 103237. 10.1016/j.midw.2021.103237 34999509

[25] Kassa SF, Alemu TG, Techane MA, Wubneh CA, Assimamaw NT, Belay GM, Tamir TT, Muhye AB, Kassie DG, Wondim A, Terefe B. The co-utilisation of oral rehydration solution and zinc for treating diarrhoea and its associated factors among under-five children in Ethiopia: further analysis of EDHS 2016. Patient Prefer Adherence. 2022;1:1713–21. 10.2147/PPA.S356557 PMC9314449 35903082

[26] Gwarzo GD. Mothers’ awareness and use of zinc in under-five children with diarrhoea in Northwestern Nigeria Nigerian. J Paediatrics. 2018;45(2):81–5.

[27] Yimenu DK, Kasahun AE, Chane M, Getachew Y, Manaye B, Kifle ZD. Assessment of knowledge, attitude, and practice of child caregivers towards oral rehydration salt and zinc for the treatment of diarrhoea in under-five children in Gondar town. Clin Epidemiol Global Health. 2022;1(14): 100998.

[28] Wang W, MacDonald VM, Paudel M, Banke KK. National scale-up of zinc promotion in Nepal: results from a post-project population-based survey. J Health Popul Nutr. 2011;29(3):207. 21766556 10.3329/jhpn.v29i3.7868 PMC3131121

[29] Thammanna PS, Sandeep M, Sridhar PV. Awareness among mothers regarding oral rehydration salt solution in the management of diarrhoea: A cross-sectional study. Indian J Child Health. 2015;2(4):215–8.

[30] Kesari A, Noel JY. Nutritional assessment.2022 35593821

[31] Uggla C, Mace R. Parental investment in child health in sub-Saharan Africa: a cross-national study of health-seeking behaviour. Royal Society open science. 2016;3(2): 150460. 26998319 10.1098/rsos.150460 PMC4785970

[32] Sen A. Missing women revisited, 2003. BMJ Br Med J. 2003. 10.1136/bmj.327.7427.1297. 10.1136/bmj.327.7427.1297 PMC286281 14656808

[33] Das M, Angeli F, Krumeich AJ, Van Schayck OC. The gendered experience with respect to health-seeking behaviour in an urban slum of Kolkata, India. Int J Equity Health. 2018;17:1–4. 29444674 10.1186/s12939-018-0738-8 PMC5813424

[34] Juma K, Juma PA, Shumba C, Otieno P, Asiki G. Non-communicable diseases and urbanisation in African cities: a narrative review. Pub Health Develop Countries-Challenges Opportunities. 2019;15(15):31–50.

[35] Caviglia M, Aringa M, Putoto G, Buson R, Pini S, Youkee D, Jambai A, Vandy MJ, Rosi P, Hubloue I, Della CF. Improving access to healthcare in Sierra Leone: the role of the newly developed national emergency medical service. Int J Environ Res Public Health. 2021;18(18):9546. 34574468 10.3390/ijerph18189546 PMC8472563

[36] Evans MV, Andréambeloson T, Randriamihaja M, Ihantamalala F, Cordier L, Cowley G, Finnegan K, Hanitriniaina F, Miller AC, Ralantomalala LM, Randriamahasoa A. Geographic barriers to care persist at the community healthcare level: evidence from rural Madagascar. PLOS Global Pub Health. 2022;2(12): e0001028. 36962826 10.1371/journal.pgph.0001028 PMC10022327

